# Toxigenic potential and antimicrobial susceptibility of *Bacillus cereus* group bacteria isolated from Tunisian foodstuffs

**DOI:** 10.1186/s12866-019-1571-y

**Published:** 2019-08-24

**Authors:** Maroua Gdoura-Ben Amor, Sophie Jan, Florence Baron, Noël Grosset, Antoine Culot, Radhouane Gdoura, Michel Gautier, Clarisse Techer

**Affiliations:** 1 Laboratory Research of Toxicology-Microbiology Environmental and Health LR17ES06, Sciences Faculty of Sfax, University of Sfax, Sfax, Tunisia; 2 Equipe Microbiologie, Agrocampus Ouest, INRA, UMR1253 Science et Technologie du Lait et de l’Œuf, Rennes, France; 3 Mixscience, Rue des Courtillons, ZAC Cissé Blossac, 35712 Bruz, France

**Keywords:** *Bacillus cereus*, Foodstuffs, Virulence genes, Cytotoxicity, Antibiotic resistance

## Abstract

**Background:**

Despite the importance of the *B. cereus* group as major foodborne pathogens that may cause diarrheal and/or emetic syndrome(s), no study in Tunisia has been conducted in order to characterize the pathogenic potential of the *B. cereus* group. The aim of this study was to assess the sanitary potential risks of 174 *B. cereus* group strains isolated from different foodstuffs by detecting and profiling virulence genes (*hblA*, *hblB, hblC*, *hblD*, *nheA*, *nheB*, *nheC*, *cytK*, *bceT* and *ces*), testing the isolates cytotoxic activity on Caco-2 cells and antimicrobial susceptibility towards 11 antibiotics.

**Results:**

The entertoxin genes detected among *B. cereus* isolates were, in decreasing order, *nheA* (98.9%), *nheC* (97.7%) and *nheB* (86.8%) versus *hblC* (54.6%), *hblD* (54.6%), *hblA* (29.9%) and *hblB* (14.9%), respectively encoding for Non-hemolytic enterotoxin *(*NHE) and Hemolysin BL *(*HBL). The isolates are multi-toxigenic, harbouring at least one gene of each NHE and HBL complexes associated or not to *bceT*, *cytK-2* and *ces* genes. Based on the incidence of virulence genes, the strains were separated into 12 toxigenic groups. Isolates positive for *cytK* (37,9%) harbored the *cytK-2 variant.* The detection rates of *bceT* and *ces* genes were 50.6 and 4%, respectively. When bacteria were incubated in BHI-YE at 30 °C for 18 h and for 5 d, 70.7 and 35% of the strains were shown to be cytotoxic to Caco-2 cells, respectively. The cytotoxicity of *B. cereus* strains depended on the food source of isolation. The presence of virulence factors is not always consistent with cytotoxicity. However, different combinations of enterotoxin genetic determinants are significantly associated to the cytotoxic potential of the bacteria. All strains were fully sensitive to rifampicin, chloramphenicol, ciprofloxacin, and gentamycin. The majority of the isolates were susceptible to streptomycin, kanamycin, erythromycin, vancomycin and tetracycline but showed resistance to ampicillin and novobiocin.

**Conclusion:**

Our results contribute data that are primary to facilitate risk assessments in order to prevent food poisoning due to *B. cereus* group.

## Background

*B. cereus* group bacteria, given their widespread nature, can be found in different types of foodstuffs. These bacteria are usually associated with two types of issues, one related to foodborne outbreaks and another one related to food spoilage. The contamination of foods with *B. cereus* group bacteria may lead to food poisoning events that usually occur under the emetic and/or the diarrheal syndromes [1]. These foodborne outbreaks are generally benign and spontaneously resolved. However, *B. cereus* group bacteria may also occasionally lead to hospitalization or even death of immunosuppressed people [2–6]. The emetic type of food poisoning is caused by the ingestion of cereulide, which is preformed in food. This toxin is a small cyclic dodecadepsipeptide encoded by the *ces* gene. The cereulide is heat and pH stable, highly resistant to protease activity and it remains active through the gastro-intestinal passage [7]. The diarrheal type of food poisoning is caused by one or several heat-labile enterotoxins that can be formed in in the small intestine. The enterotoxins produced by *B*. *cereus* group bacteria that are recognized as playing a major role in the diarrheal disease are the Hemolysin BL (HBL) encoded by *hblA, hblB, hblC,* and *hblD*; the Non-Hemolytic Enterotoxin (NHE) encoded by *nheA, nheB* and *nheC*, and the Cytotoxin K (CytK) encoded by *cytK* [8, 9]. Two CytK variants encoded by *cytK-1* and *cytK-2* genes, have been described by Guinebretière et al. [10] and Castiaux et al. [11]. CytK-1 shows 89% protein sequence homology with that of CytK-2, but carries much higher toxicity.

Apart from HBL, NHE and CytK, also Enterotoxin T that is encoded by the *bceT* gene, belongs to the group of diarrhoeal enterotoxins. Contribution to food poisoning of BceT enterotoxin [12], could never be confirmed and as a result of later studies the reported activity and identity of BceT as entertoxin is questionable [13, 14]. It was suggested that the *bceT* gene product does not possess biological activity and cannot contribute to outbreaks [13], and seems to be a cloning artifact [14].

The actual risk of food poisoning due to the *B. cereus* group depends on the level of expression of the virulence genes [15–18]. The emetic and the diarrheal syndromes can occur when the bacterial cell concentration reaches a level of 5 to 8 log_10_ CFU/g and of 5 to 7 log_10_ CFU/g, respectively [19, 20]. Therefore, it is generally advised to food industries that foods with 10^5^ CFU/g of *B. cereus* are considered unsafe for consumption [21].

With the aim to better evaluate the in vivo conditions of toxinogenesis, several studies assessed the cytotoxicity of *B. cereus* strains on CHO, Vero, Hep-2 or Caco-2 cells [22–25]. In recent years, the accelerated emergence of foodborne pathogens resistant to a variety of antibiotics is one of the most serious threats for public health and clinical perspectives, which can cause perturbation in the empirical therapy during outbreaks. Many previous reports have shown that *B. cereus* group bacteria isolated from different foods are resistant to several antibiotics such as ampicillin, penicillin streptomycin, tetracycline, trimethoprim, and ceftriaxone [26–29]. Therefore, it is important to evaluate the resistance of foodborne *B. cereus* group bacteria to a variety of antibiotics for a better management of infectious diseases. The objective of the present work was to investigate the toxigenic potential of a collection of 174 *B. cereus* group strains coming from Tunisian foodstuffs, (i) by detecting the presence of virulence genes, (ii) by assaying the cytotoxic activity of bacterial supernatants on Caco-2 cells, and (iii) by assessing their antimicrobial resistance pattern towards selected antibiotics.

## Methods

### Bacterial isolation and identification

The collection analysed comprised 174 *B. cereus* group strains. They were previously isolated from 687 Tunisian food samples (cereals, spices, cooked food, canned products, seafood products, dairy products, fresh-cut vegetables, raw and cooked poultry meats), collected randomly from supermarkets, hotels, restaurants and private companies during the period from April 2014 to April 2015 [30]. Ten grams of each food sample **were** homogenized for 1 min with 90 ml of buffered peptone water (VWR, Strasbourg, France) containing 5 g/l of lithium chloride (Prolabo, Fontenay sur bois, France) in a BagMixer stomacher (AES Laboratory, Combourg, France). After serial dilution, 0.1 ml of each diluted sample was streaked in Mannitol Egg Yolk Polymixin agar medium (MYP) (Oxoid, Basingstoke, England) and plates were incubated for 24 h at 30 °C. The presumptive identification of *B. cereus* group bacteria was based on the appearance of rough colonies with a violet-red background, and surrounded by a white egg yolk precipitate. One typical colony from each sample was subcultured and preserved as cryoculture at − 80 °C after addition of glycerol (Sigma Aldrich, Saint Quentin Fallavier, France) at a final concentration of 25%. To verify whether *B. cereus*-like isolates belonged to the *B. cereus* group, a PCR test targeting the sspE gene sequence specific of the group was carried out [31].

### DNA extraction

Extraction of DNA was performed according to the Chelex extraction method [32]. Briefly, after twice overnight propagation of each frozen isolate in BHI-YE (Fisher Bioblock, Illkirch, France) at 30 °C without agitation, 5 ml of each culture was transferred into à 15 ml falcon tube containing 300 μl of 25% (m/v) sterile suspension of Chelex beads (Grosseron, Saint-Herblain, France) prepared in sterile Milli-Q water (Sigma Aldrich). Mixture was vortexed and centrifuged at 7000 rpm for 7 min at 4 °C. The cell pellet was resuspended in 200 μl sterile Milli-Q water (Sigma Aldrich) and lysed by heating at 100 °C for 10 min. After centrifugation at 7000 rpm at 4 °C for 7 min, 150 μl of supernatant was collected and re-centrifuged under the same conditions. The concentration of DNA was determined with a NanoDrop ND − 1000 spectrophotometer (Nanodrop Technologies, Wilmington, USA) and the sample was diluted to a final concentration of approximately 100 ng/μl.

### Detection of virulence genes

Confirmed *B. cereus* group strains were screened for the emetic (*ces*) and the enterotoxigenic genes (*hblA*, *B*, *C*, and *D*, *nheA*, *B*, and *C*, *bceT* and *cytK* and its variants *cytK-1* and *cytK-2*) genes. All primers used as well as their annealing temperatures and the size of the amplified fragment for each gene are shown in Table 1. The detection of the *ces* cluster was tested with two different primer pairs [34, 35]. PCR amplification was systematically performed in a 28 μl reaction volume. Each reaction mixture contained 5 μl of 100 ng DNA template, 2 μl of each primer (Sigma Aldrich) with a concentration of 10 μM, 0.3 μl of Taq polymerase (5000 U/ml) (Biolabs, Evry, France), 0.5 μl of 10 mM deoxyribonucleotide triphosphate (Eurogentec, Seraing, Belgium), 1.12 μl of 50 mM MgCl_2_ (Biolabs), 2.5 μl of 10X AmpliTaq buffer (Biolabs) and 14.5 μl of sterile Milli-Q water (Sigma Aldrich). The amplification reactions were carried out in a PCR thermocycler (iCycler optical module 584BR; Bio-Rad, Marnes-la-Coquette, France). For the *ces* cluster, the amplification conditions were 5 min at 95 °C, followed by 30 cycles of 15 s at 95 °C, 30 s at 58 °C and 30 s at 72 °C and a final extension at 72 °C for 8 min. For *hblB*, the amplification conditions were 2 min at 94 °C followed by 10 cycles of 10 s at 94 °C, 30 s at 58 °C, and 2 min at 68 °C. The ten cycles were followed by 20 cycles of 10 s at 94 °C, 30 s at 58 °C, and 2 min (plus 20 s per cycle) at 68 °C; and a final extension at 68 °C for 7 min [33]. For the remaining toxin genes (*hblA*, *hblC*, *hblD*, *nheA*, *nheB*, *nheC*, *bceT* and *cytK* and its variants *cytK-1* and *cytK-2*), the amplification conditions were 4 min at 95 °C followed by 30 cycles of 30 s at 95 °C, 30 s at the annealing temperatures (Table 1) and 1 min at 72 °C, and a final extension of 7 min at 72 °C. For each run, the whole PCR mix without any DNA template was used as a negative control. The positive controls for PCR amplification of virulence genes were the same as the ones used in Techer et al. study [36]. The mesophilic strain (TIAC 1095), harbouring the *ces* cluster, isolated from a Belgian emetic food poisoning event was used as positive control for PCR amplification of the emetic toxin gene. The strain C43, isolated from a food product [33], harbours *hblA*, *hblB*, *hblC*, *hblD*, *nheA*, *nheB*, *nheC*, and *cytK*, and was used as positive control for PCR amplification of enterotoxigenic genes.

**Table 1:** Primers used in the simplex PCR for the detection of virulence genes in *B. cereus*

Targeted Gene	Primer name	Sequence (5′- 3′)	Product Size (bp)	Annealing Temp (°C)	Reference
*hblA*	HA F HA R	AAGCAATGGAATACAATGGG AGAATCTAAATCATGCCACTGC	1154	56	[33]
*hblB*	HA F HB R	AAGCAATGGAATACAATGGG AATATGTCCCAGTACACCCG	2684	58	[33]
*hblC*	HC F HC R	GATACTCAATGTGGCAACTGC TTGAGACTGCTCGTCTAGTTG	740	58	[33]
*hblD*	HD F HD R	ACCGGTAACACTATTCATGC GAGTCCATATGCTTAGATGC	829	58	[33]
*nheA*	NA F NA R	GTTAGGATCACAATCACCGC ACGAATGTAATTTGAGTCGC	755	56	[33]
*nheB*	NB F NB R	TTTAGTAGTGGATCTGTACGC TTAATGTTCGTTAATCCTGC	743	54	[33]
*nheC*	NC F NC R	TGGATTCCAAGATGTAACG ATTACGACTTCTGCTTGTGC	683	54	[33]
*bceT*	bceT-f bceT-r	GCTACGCAAAAACCGAGTGGTG AATGCTCCGGACTATGCTGACG	679	57	[12]
*cytK*	CK F CK R	ACAGATATCGG(G,T)CAAAATGC TCCAACCCAGTT(A,T)(G,C) CAGTTC	809	54	[11]
*cytK-1*	CK1 F CK1 R	CAATTCCAGGGGCAAGTGTC CCTCGTGCATCTGTTTCATGAG	426	57	[11]
*cytK-2*	CK2 F CK2 R	CAATCCCTGGCGCTAGTGCA GTGIAGCCTGGACGAAGTTGG	585	57	[11]
*ces*	EM1F EM1R	GACAAGAGAAATTTCTACGAGCAAGTAAT GCAGCCTTCCAATTACTCCTTCTGCCACAGT	635	58	[34]
	CesF1 CesR2	GGTGACACATTATCATATAAGGTG GTAAGCGAACCTGTCTGTAACAACA	1271	58	[35]

### Cytotoxic activity

Cytotoxic activity of bacterial supernatants on Caco-2 cells were performed according to Jan et al. [24]. Briefly, after a defrosting step, Caco-2 cells were cultivated on 96-well microplates at 37 °C under 5% CO_2_ atmosphere for 3 to 4 d in Dulbecco modified Eagle medium (Sigma Aldrich) supplemented with 10% (v: v) fetal calf serum (Cambrex, North Brunswick, N.J.), 100 UI/ml penicillin (Sigma Aldrich), 100 μg/ml streptomycin (Sigma Aldrich) and 2.5 μg/ml amphotericin B (Sigma Aldrich). Bacteria were grown for 18 h or 5 d at 30 °C in BHI-YE, without agitation (Fisher Bioblock). After centrifugation (10 min, 7000 rpm, 4 °C), the supernatants were filtered through 0.2 μm sterile filter units (Starstedt, Nümbrecht, Germany). After removal of the culture medium, Caco-2 cells were washed 3 times with phosphate buffered saline (PBS) (Gibco, Paisley, UK), incubated during 3 h with 50 μl of each bacterial filtrate and then rinsed with PBS (Gibco) and fixed with 2% (w: v in PBS) paraformaldehyde (Sigma Aldrich) at 4 °C for 30 min. After removal of the paraformaldehyde, the remaining cells were stained for 20 min at room temperature with 80 μl of crystal violet solution (Sigma Aldrich,). Cells were rinsed three times with distilled water, and the crystal violet solution was released from the cells by adding 200 μl of 50% (v: v) ethanol in water and shaking the microplates at room temperature for 45 min. After transfer into new microplates, the amount of released dye was measured at 630 nm and was inversely related to the cytotoxic activity of culture filtrates. The cytotoxic activity was expressed as a percentage of inhibition compared with the control (BHI-YE alone), calculated as follows: (Optical Density (OD) control – OD assay)/OD control × 100. Filtrates were considered cytotoxic whenever the OD represented less than 50% of that of the control (percentage of inhibition higher than 50%). Tests and controls were done in triplicate on the same microplate.

### Antibiotic susceptibility testing

The antibiotic susceptibility of the isolates was studied using the Kirby–Bauer disk diffusion method [37]. Mueller-Hinton Agar (Merck, Darmstadt, Germany) was used for this test. All isolates were grown in BHI-YE (Fisher Bioblock) for 24 h at 30 °C, without agitation, followed by spreading on Mueller-Hinton agar plates. Eleven antimicrobials were chosen for antibiotic sensitivity testing, including ampicillin (10 μg), vancomycin (30 μg), gentamycin (10 μg), erythromycin (15 μg), tetracycline (30 μg), ciprofloxacin (5 μg), chloramphenicol (30 μg), novobiocin (30 μg), streptomycin (10 μg), kanamycin (30 μg) and rifampicin (5 μg). All Muller-Hinton plates were incubated at 30 °C for 18–24 h. The inhibition zones were measured and interpreted referring to the Clinical and Laboratory Standards Institute (CLSI) [38], which contains measurement ranges and their equivalent qualitative categories of susceptible, intermediately susceptible or resistant.

### Statistical analyses

The statistical analyses, including *t*-tests, and ANOVA *F*-test, were performed using the R - 3.4.2. statistic software. A *p*-value < 0.05 was considered as statistically significant for all the parameters evaluated. The *F*-test was used to assess the potential relationship between (i) the origin of the strains and their cytotoxity and (ii) the type of virulence factors they harbour and their level of cytotoxity. The *t*-tests were used to assess the level of cytotoxicity of each strain inside the collection as well as the potential correlation between the type of virulence factors they harbour and their level of cytotoxicity.

## Results

### Distribution of enterotoxin and emetic toxin-encoding genes among *B. cereus* collection

In order to characterize the virulence potential of foodborne *B. cereus* group bacteria in Tunisia, 174 isolates from different kinds of foods were screened by PCR for the presence of nine diarrhoeal toxin-encoding genes (*hblABCD* complex, *nheABC* complex, *bceT, and cytK* and its variants) and one emetic toxin-encoding gene (*ces*). At least one gene of each NHE and HBL complexes was detected in 100 and 59.2% of strains, respectively. The enterotoxin genes detected among *B. cereus* isolates were, in decreasing order, *nheA* (98.9%), *nheC* (97.7%) and *nheB* (86.8%) versus *hblC* (54.6%), *hblD* (54.6%), *hblA* (29.9%) and *hblB* (14.9%), respectively for the NHE and HBL complexes.

The genetic determinants of the NHE complex were shown to be the most common genes detected inside the collection. All three genes of the NHE complex were detected in 84.5% (147/174) of the collection. The presence of two genes was observed in 14.4% (25/174) of the collection, while 1.1% (2/174) of the collection harboured only one gene (Table 2). The four genes encoding the HBL complex were detected in 13.8% (24/174) of the collection, three genes were present in 22.4% (39/174) of the collection; 14.4% (25/174) were positive for two genes, 8.6% (15/174) had a single gene of the complex, while 40.8% (71/174) had no HBL genes (Table 2). The *cytK* gene was present in 37.9% of the strains but further testing revealed that strains harbouring the *cytK* gene belonged to the *cytK-2* type. None of the *B. cereus* strains haboured the *cytK-1* variant.

**Table 2:** Total distribution of virulence genes in *B. cereus* strains collection (*n* = 174) isolated from foodstuffs in Tunisia

Toxigenic genes	No. (%) of strains positive for target gene(s)										
	Cooked food (*n* = 42)	Pastry products (*n* = 37)	Cereal products (*n* = 23)	Cooked poultry meat (*n* = 18)	Spices (n = 17)	Seafood products (*n* = 11)	Canned products (*n* = 9)	Raw poultry meat (*n* = 8)	Fresh-cut vegetables (*n* = 5)	Dairy products (n = 4)	Total (n = 174)
HBL gene complexes											
*hblC*	1 (2.4)	4 (10.8)	1 (4.3)	1(5.6)	0 (0.0)	0 (0.0)	0 (0.0)	0 (0.0)	0 (0.0)	0 (0.0)	7 (4.0)
*hblD*	3 (7.1)	0 (0.0)	1(4.3)	0 (0.0)	1 (5.9)	1 (9.1)	0 (0.0)	2 (25.0)	0 (0.0)	0 (0.0)	8 (4.6)
*hblA*+ *hblC*	1 (2.4)	0 (0.0)	0 (0.0)	0 (0.0)	0 (0.0)	0 (0.0)	0 (0.0)	0 (0.0)	0 (0.0)	0 (0.0)	1 (0.6)
*hblC*+ *hblD*	7 (16.7)	8 (21.6)	2 (8.7)	0 (0.0)	0 (0.0)	1 (9.1)	2 (22.2)	0 (0.0)	3 (60.0)	1 (25.0)	24 (13.8)
*hblA*+ *hblC*+ *hblD*	5 (11.9)	4(10.8)	2 (8.7)	3 (16.7)	7 (41.2)	4 (36.4)	0 (0.0)	1 (12.5)	1 (20.0)	0 (0.0)	27 (15.5)
*hblB*+ *hblC*+ *hblD*	2 (4.8)	3 (8.1)	1(4.3)	2 (11.1)	1 (5.9)	1 (9.1)	1 (11.1)	1 (12.5)	0 (0.0)	0 (0.0)	12 (6.9)
*hblA*+ *hblB*+ *hblC*+ *hblD*	6 (14.3)	8 (21.6)	5 (21.8)	2(11.1)	3 (17.6)	0 (0.0)	0 (0.0)	0 (0.0)	0 (0.0)	0 (0.0)	24 (13.8)
None detected	17 (40.4)	10 (27.0)	11 (47.8)	10 (55.6)	5 (29.4)	4 (36.4)	6 (66.7)	4 (50.0)	1 (20.0)	3 (75.0)	71 (40.8)
NHE gene complexes											
*nheA*	1 (2.4)	0 (0.0)	0 (0.0)	0 (0.0)	0 (0.0)	0 (0.0)	0 (0.0)	0 (0.0)	0 (0.0)	0 (0.0)	1 (0.6)
*nheB*	0 (0.0)	0 (0.0)	0 (0.0)	0 (0.0)	1 (5.9)	0 (0.0)	0 (0.0)	0 (0.0)	0 (0.0)	0 (0.0)	1 (0.6)
*nheA*+ *nheB*	9 (21.4)	4(10.8)	1(4.3)	1(5.6)	1 (5.9)	2 (18.2)	0 (0.0)	3 (37.5)	0 (0.0)	1 (25.0)	22 (12.6)
*NheA*+ *nheC*	2 (4.8)	0 (0.0)	0 (0.0)	0 (0.0)	0 (0.0)	0 (0.0)	0 (0.0)	0 (0.0)	0 (0.0)	0 (0.0)	2 (1.1)
*nheB*+ *nheC*	1 (2.4)	0 (0.0)	0 (0.0)	0 (0.0)	0 (0.0)	0 (0.0)	0 (0.0)	0 (0.0)	0 (0.0)	0 (0.0)	1 (0.6)
*nheA*+ *nheB*+ *nheC*	29 (69.0)	33 (89.2)	22 (95.7)	17 (94.4)	15 (88.2)	9 (81.8)	9 (100.0)	5 (62.5)	5 (100.0)	3 (75.0)	147 (84.5)
None detected	0 (0.0)	0 (0.0)	0 (0.0)	0 (0.0)	0 (0.0)	0 (0.0)	0 (0.0)	0 (0.0)	0 (0.0)	0 (0.0)	0 (0.0)
Other genes											
*cytK*	13 (30.1)	15 (40.54)	9 (39.1)	6 (33.3)	9 (52.9)	8 (72.7)	3 (33.3)	1 (12.5)	1 (20.0)	1 (25.0)	66 (37.9)
*bceT*	17 (40.4)	20 (54.1)	14 (60.9)	9 (50.0)	10 (58.8)	10 (90.9)	2 (22.2)	5 (62.5)	1 (20.0)	0 (0.0)	88 (50.6)
*ces*	2 (4.8)	1 (2.7)	0 (0.0)	3 (16.7)	1 (5.9)	0 (0.0)	0 (0.0)	0 (0.0)	0 (0.0)	0 (0.0)	7 (4.0)

The *bceT* and *ces* genes were present in 50.6 and 4% of the collection, respectively (Table 2).

The virulence genes were widely distributed regardless the origin of the strains, except for the emetic toxin-encoding gene (*ces*) that was only detected in the strains coming from cooked poultry meat, cooked food, pastry products and spices. All isolates presented at least one of the genes investigated. The virulence genes distribution revealed a toxigenic diversity among *B. cereus* group isolates (Table 3). Twelve groups (G1 to G12) are compiled in Table 3.

**Table 3:** Distribution of different combinations of virulence genes in *B. cereus* group isolates in different groups

Group	Genes presents	Number of isolates	% isolates in each group
G1	HBL complex, NHE complex, *bceT*, *cytK*	36(8+ 26^a^ + 1^b^ + 1^c^)	20.7
G2	HBL complex, NHE complex, *bceT*	29(9+ 15^a^ + 1^b^ + 4^c^)	16.7
G3	HBL complex, NHE complex, *cytK*	7(1+ 3^a^ + 3^c^)	4.0
G4	HBL complex, NHE complex	30(4+ 16^a^ + 10^c^)	17.2
G5	NHE complex, *bceT*, *cytK*	8	4.6
G6	NHE complex, *bceT*	13(10+ 3^b^)	7.5
G7	NHE complex, *cytK*	12(11+ 1^b^)	6.9
G8	NHE complex	32(30+ 2^b^)	18.4
G9	NHE complex, HBL complex, *ces*	1^c^	0.6
G10	NHE complex, *cytK*, *ces*	3	1.7
G11	NHE complex, *bceT*, *ces*	2	1.1
G12	NHE complex, *ces*	1	0.6

^a^Lacked at least one gene of HBL complex; ^b^Lacked at least one gene of NHE complex; ^c^Lacked at least one gene of NHE & HBL complex

### Cytotoxic activity of *B. cereus* strains on Caco-2 cells

Each of the 174 isolates was able to grow in BHI-YE. The average bacterial population was 8 ± 0.3 and 7 ± 0.5 log_10_ CFU/ ml after 18 h and 5 d incubation at 30 °C, respectively (results not shown). The percentage of cytotoxic strains observed after 18 h incubation (70.7%) was twice the one observed after 5 d incubation (35%) in BHI-YE at 30 °C (results not shown).

#### Cytotoxicity and strains origins

A significant correlation is found regarding the cytotoxicity and the source of strains (*F*- test, *p* < 0.05). As compared to strains isolated from spices, a significant dispersion of cytotoxic activity has been reported for strains isolated from fresh cut vegetables, cereals or dairy products (*p* < 0.05). Cytotoxic activity was comparable in all strains when isolated from cooked and raw poultry meat, canned products, pastry products and cooked foods (*p* > 0.05). For strains isolated from seafood and spices, the level of cytotoxicity was significantly influenced by the incubation time of the bacterial culture (*p* = 0.0004). After 18 h of incubation, a significant difference in cytotoxic activity was observed. However, after 5 days of incubation, cytotoxicity was found to be comparable (*p* > 0.05, *p* = 0.09) (results not shown).

#### Cytotoxicity and virulence gene profiles

The involvement of several known virulence factors in the cytotoxicity on Caco-2 cells was evaluated. The presence of the HBL genetic determinants was not related to the cytotoxicity. Indeed, among strains possessing or not possessing the four genetic determinants of the HBL complex, cytotoxic and non-cytotoxic strains were found. The percentage of cytotoxic strains harbouring the four genes was higher after 18 h than after 5 d incubation. Therefore, among the 24 strains possessing the whole HBL genetic determinants, 79.2 and 41.7% displayed toxicity on Caco-2 cells after 18 h and 5 d incubation, respectively. Although 147 strains carried the three genetic determinants of the NHE complex, only 68.7 and 36.7% of them were shown to be cytotoxic after 18 h and 5 d incubation, respectively. Moreover, among the strains that lacked one or two of the genetic determinants of the NHE complex, both cytotoxic and non-cytotoxic strains were highlighted at both time of incubation.

Several combinations of NHE and HBL genetic determinants were significantly associated (*t*-test, *p* < 0.05) with the cytotoxic activity of culture supernatants (Table 4). The level of cytotoxicity increased significantly (+ 12% and + 14% after 18 h and 5 d incubation, respectively) when strains harbored simultaneously *hblC* and *nheC*. The presence of *hblC* and *nheB* had a significant positive incidence on the cytotoxicity (+ 27% and + 23% after 18 h and 5 d, respectively). However, the presence of *nheB* and the absence of *hblC* was associated with a decrease of the cytotoxicity after 5 d incubation (− 17%). The simultaneous presence of *hblB* and *nheA* significantly correlated with the cytotoxic activity after 5 d incubation (+ 11%). The cytotoxity of supernatent after 5 d incubation decrease by 29% when strains harbored *hblB* but not *nheA*. The simultaneous carriage of *hblD* and *nheC* was inversely associated with cytotoxicity. It decreased by 8% and − 14% after 18 h and 5 d incubation, respectively.

**Table 4:** Statistical analyses of associations between the presence or absence of virulence factors and cytotoxicity on Caco-2 cells

*F*-test					*t*-test				
Significant association tested Cytotoxicity versus virulence factors	Sum Sq		*p*-value		Significant association tested Cytotoxicity versus Presence^b^/ Absence^a^ of virulence factors	Estimate		*p*-value	
	Cytotoxicity after		Cytotoxicity after			Cytotoxicity after		Cytotoxicity after	
	18 h	5d	18 h	5d		18 h	5d	18 h	5d
*hblC:hblD*	5562	20,994	0.0032 **	4.57E-08 ***	*hblC* ^*b*^ *:hblD* ^*b*^	ND	14	ND	4.75E-08 ***
					*hblC* ^*a*^ *:hblD* ^*b*^	-10	ND	0.0034 *	ND
*hblC:nheB*	13,568	10,441	1.58E-07 ***	0.0005 ***	*hblC* ^*b*^ *:nheB* ^*b*^	27	24	1.58E-07 ***	0.0073 **
					*hblC* ^*a*^ *:nheB* ^*b*^	ND	−17	ND	0.0058 **
*hblC:nheC*	6497	11,695	0.0002 ***	4.08E-05 ***	*hblC* ^*b*^ *:nheC* ^*b*^	12	14	0.0003 ***	4.08E-05 ***
*hblD:nheC*	3675	11,106	0.0058 **	6.33E-05 ***	*hblD* ^*b*^ *:nheC* ^*b*^	−8	−14	0.0058 **	6.33E-05 ***
*bceT: hblB*	23,036	8048	1.12E-10 ***	0.0006 ***	*bceT* ^*b*^ *:hblB* ^*b*^	18	10	0.0002 ***	0.0006 ***
					*bceT* ^*a*^ *:hblB* ^*b*^	−16	ND	3.07E-07 ***	ND
*nheC:bceT:hblB*	11,787	6560	6.14E-06 ***	0.0085 **	*nheC* ^*b*^ *:bceT* ^*b*^ *:hblB* ^*b*^	−17	ND	0.0004 ***	ND
					*nheC* ^*b*^ *:bceT* ^*a*^ *:hblB* ^*b*^	9	ND	0.004 **	ND
					*nheC* ^*b*^ *:bceT* ^*b*^ *:hblB* ^*a*^	ND	6	ND	0.012 *
*bceT:hblD*	11,867	ND	8.99E-07 ***	ND	*bceT* ^*b*^ *:hblD* ^*b*^	−6	ND	8.99E-07 ***	ND
*bceT:nheC*	4733	ND	0.0017 **	ND	*bceT* ^*b*^ *:nheC* ^*b*^	−12	ND	0.0017 **	ND
*ces:hblC*	9828	ND	7.46E-06 ***	ND	*ces* ^*b*^ *:hblC* ^*b*^	−17	ND	7.46E-06 ***	ND
*cytK:hblD*	19,045	ND	6.51E-10 ***	ND	*cytK* ^*b*^ *:hblD* ^*b*^	14	ND	6.51E-10 ***	ND
*cytK:nheC*	6617	ND	0.0002 ***	ND	*cytK* ^*b*^ *:nheC* ^*b*^	−12	ND	0.0002 ***	ND
*cytK:nheC:hblB*	4501	ND	0.0095 **	ND	*cytK* ^*b*^ *:nheC* ^*a*^ *:hblB* ^*b*^	12	ND	0.011 *	ND
*hblC:cytK*	15,874	ND	1.53E-08 ***	ND	*hblC* ^*b*^ *:cytK* ^*b*^	−12	ND	1.53E-08 ***	ND
*hblD:hblA*	2417	ND	0.025 *	ND	*hblD* ^*b*^ *:hblA* ^*b*^	−8	ND	0.025 *	ND
*nheB:nheC*	6500	ND	0.0012 **	ND	*nheB* ^*a*^ *:nheC* ^*b*^	27	ND	0.01 *	ND
					*nheB* ^*b*^ *:nheC* ^*b*^	−9	ND	0.018 *	ND
*bceT:hblA*	ND	8530	ND	0.002 **	*bceT* ^*b*^ *:hblA* ^*b*^	ND	−7	ND	0.0008 ***
*hblC:hblB*	ND	5000	ND	0.007 **	*hblC* ^*b*^ *:hblB* ^*b*^	ND	−11	ND	0.007 **
*nheA:hblB*	ND	8433	ND	0.002 **	*nheA* ^*a*^ *:hblB* ^*b*^	ND	−29	ND	0.047 *
					*nheA* ^*b*^ *:hblB* ^*b*^	ND	11	ND	0.025 *
*cytK:hblA*	3400	ND	0.029 *	ND	*cytK* ^*b*^ *:hblA* ^*b*^	NS	ND	> 0.05	ND
					*cytK* ^*a*^ *:hblA* ^*b*^	NS	ND	> 0.05	ND
*cytK:nheC:hblA*	3165	ND	0.0375 *	ND	*cytK* ^*b*^ *:nheC* ^*b*^ *:hblA* ^*b*^	NS	ND	> 0.05	ND
					*cytK* ^*a*^ *:nheC* ^*b*^ *:hblA* ^*b*^	NS	ND	> 0.05	ND

^a^absence; ^b^presence; *ND* Not determined; *NS* Not significant (*p*-value≥0.05); *: significant (*p*-value: 0.01 to 0.05); **: very significant (*p*-value: 0.001 to 0.01); ***: very significant (*p*-value < 0.001)

Among the isolates that possess the *cytK* gene, there were both cytotoxic and non-cytotoxic strains after 18 h and 5 d incubation. As shown in Table 4, a significant correlation was shown between cytotoxicity and the combination of *cytK*: *hblD* as well as with the combination of *cytK*: *hblB* with the absence of *nheC,* after 18 h incubation. Both *cytK*: *nheC* and *cytK: hblC* combinations were inversely correlated with the cytotoxic activity after 18 h incubation.

*bceT* was present in cytotoxic strains as well as in safe strains. The simultaneous presence of *bceT*, *nheC* and *hblB* was significantly associated with a lower cytotoxic activity after 18 h (− 17%) (Table 4). However, when *bceT* was present with *nheC* or *hblD*, the cytotoxicity after 18 h incubation decreased by 12 and 6%, respectively. After 18 h incubation, the cytotoxicity increased significantly (18%) when strains harbored *bceT* and *hblB*. When strains carried *hblB* but lacked *bceT*, the cytotoxicity decreased significantly by 16% (Table 4). The absence of *bceT* was associated with a higher cytotoxic activity after 18 h (+ 9%) when strains harbored simultaneously *nheC* and *hblB*, whereas it was lower (− 17%) when strains harbored simultaneously *nheC*, *hblB* and *nheC* and *hblB.*

After 5 d, the cytotoxicity was higher when strains possessed simultaneously *bceT* and *hblB* (+ 10%) and when carried *bceT* and *nheC* but not *hblB* (+ 6%) (Table 4). However, the cytotoxicity was lower when strains harbored simultaneously *bceT* and *hblA* (− 7%).

All the strains harbouring the *ces* gene have cytotoxic activity against Caco-2 cells after 18 h incubation. When *ces* was associated with *hblC*, the cytotoxicity was significantly lower (− 17%) (Table 4). Moreover, no cytotoxic activity was found for these strains after 5 d incubation.

### Antibiotic susceptibility

The susceptibility of 174 *B. cereus* isolates was tested for 11 different antibiotics. The results of the antimicrobial tests are presented in Table 5. All *B. cereus* strains were fully sensitive to rifampicin, chloramphenicol, ciprofloxacin, and gentamycin. The majority of the isolates were susceptible to streptomycin (98.9%), kanamycin (96.6%), erythromycin (95.4%), vancomycin (92%) and tetracycline (85.1%). In addition, all isolates were resistant to ampicillin (90.8%) and novobiocin (88%).

**Table 5:** Antibiotic susceptibility of 174 *B. cereus* strains isolated from foodstuffs in Tunisia

Antibiotics	Conc. (μg/disc)	No. of strains (%)		
		Resistant	Intermediate	Susceptible
Rifampicin	5	0	0	174 (100)
Erythromycin	15	2 (1.2)	6 (3.4)	166 (95.4)
Chloramphenicol	30	0	0	174 (100)
Novobiocin	30	153 (88)	13 (7.5)	8 (4.5)
Ampicillin	10	158 (90.8)	11 (6.3)	5 (2.9)
Ciprofloxacin	5	0	0	174 (100)
Streptomycin	10	2 (1.1)	0	172 (98.9)
Gentamycin	10	0	0	174 (100)
Vancomycin	30	7 (4)	7 (4)	160 (92)
Kanamycin	30	0	6 (3.4)	168 (96.6)
Tetracycline	30	10 (5.7)	16 (9.2)	148 (85.1)

## Discussion

This study revealed the toxigenic potential of *B. cereus* group strains collection isolated from different kinds of foodstuffs collected in Tunisia. The pathogenic abilities of *B. cereus* group strains were evaluated by examination of the presence of virulence factors, the cytotoxic activity and the antimicrobial resistance.

The isolates are multi-toxigenic, harbouring at least one gene of each NHE and HBL complexes associated or not to *bceT*, *cytK-2* and *ces* genes*.* The inability to detect all genes by PCR in most isolates is due to the existence of a polymorphism in the sequences of HBL and NHE complexes genes rather than their absence [33]. According to our results, the genes of the HBL complex were less common than that of the NHE complex. The low prevalence of genetic determinants of the HBL complex compared to those of the NHE complex has been demonstrated in previous studies [22, 39–41]. The incidence of *nhe* and *hbl* was within the ranges described by Ceuppens et al. [15]. They reported that 84 to 100% of *B. cereus* group strains possessed *nhe* while *hbl* was detected in 29 to 92% of the isolates they studied. All (100%) the *B. cereus* group strains harbored at least one gene of the NHE complex. One hundred and three isolates (59.2%) carried at least one of the HBL genetic determinants. Similarly, Tewari et al. [39] reported that 55.2 and 89.7% of the *B. cereus* strains they studied were positive for at least one of the HBL and NHE genetic determinants, respectively. These results are in contrast to those of Ngamwongsatit et al. [42] who reported that none of the 411 *B. cereus* group strains they studied showed the presence of only a single or two genes in either the HBL or NHE complexes. *cytK* was detected in 37.9% of the collection. A similar occurrence was observed for strains isolated from cooked chilled food and vegetables [33], meat products [39] and cereal products “sunsik” [43]. However, the occurrence reaches up to 77% for strains isolated from cereal products, “sunsik” [44], and ready to eat meals, spices, dairy products, starches and flours [45]. The percentage of strains carrying *bceT* (50.6%) was in agreement with the results of Bonerba et al. [46], who demonstrated that 52% of the isolates coming from pastries, rice samples, potato meals, mozzarella and meat meals possessed this gene. According to Guinebretière, et al. [33], *bceT* appears as widely distributed in food-borne strains isolated from cooked chilled food and vegetables (71%), whereas a low occurrence of this gene was highlighted by Yang et al. [47] in food-related *B. cereus* group isolates (11.8%). As stated above, the occurrence of enterotoxin genes varies greatly depending on the study. This variability may be ascribed to various geographical locations, various sources of strains and the use of various primers for PCR assays.

Among all the strains tested in this study, only seven strains (4%) harboured the *ces* gene. These strains were detected in cooked poultry products, pastry products, cooked food and spices. The emetic intoxication is frequently associated with starchy foods such as rice, noodles, pasta and mashed potato [3, 48, 49]. Furthermore, similarly to our findings, López et al. [50] and Messelhäusser et al. [51] reported that emetic *B. cereus* group strains were detected in cooked chicken and foods such as soups, sauces, and mixed or buffet meals. However, none of the *B. cereus* group strains isolated from spices possessed the emetic toxin gene (*ces*) in the studies of Hariram and Labbé [52] and of Fogele et al. [53]. Therefore, the prevalence of emetic *B. cereus* group bacteria in different types of foods need to be further investigated in order to decipher the potential contamination sources.

Based on the incidence of all the virulence genes (*hblABCD*, *nheABC*, *cytK*, *bceT* and *ces*), *B. cereus* group strains were divided into 12 different groups (Table 3). This finding indicates that the *B. cereus* group is likely diverse in food. The detection at least of one gene from the HBL and/or NHE complex indicates the presence of both *nhe* and *hbl* operons [54]. The polymorphism among HBL and NHE complexes is the likely explanation of the failure to identify all genes in most *B. cereus* isolates by PCR [33]*.* Therfore, when strains carried at least one toxin gene, this later could be a target marker for screening toxigenic *B. cereus* group strains in food.

Emetic *B. cereus* group strains were heterologous at a genotypic level. They belonged to different toxigenic groups (G9- G12). The emetic *B. cereus* group strain belonging to the G12 group carried NHE complex genes and not those of the HBL complex. The virulence profile of these strains is consistent with the studies of Ehling-Schulz et al. [35], Lee et al. [43] and Yang et al. [55], where an absence of correlation was observed between the presence of the *ces* gene and of the HBL complex that is common in non-emetic strains.

Noteworthy, the strains belonging to the G9 group possessed the *ces* gene together with incomplete NHE and HBL complexes. Our finding is in agreement with previous studies that detected emetic strains positive for both *ces* and HBL complex genes [56, 57].

In addition to the NHE complex and the *ces* genes, the emetic strains belonging to the G10 and G11 groups possessed *cytK and bceT*, respectively. Similarly, previous studies [55, 58] reported that emetic strains harboured *cytK* or *bceT*. Thus, emetic *B. cereus* group strains containing various enterotoxin genes such as those of the NHE and HBL complexes, *cytK* and/or *bceT* could have the potential to cause diarrheal and emetic food poisoning simultaneously.

The majority of the *B. cereus* strains of the collection (70.7%) was cytotoxic after 18 h of incubation at 30 °C. However, the cytotoxicity decreased after 5 d incubation. Only 35% of strains were found cytotoxic at this time of culture. The fact that the percentage of cytotoxic strains was lower after 5 d of incubation than after 18 h was probably the result of dying of cells [24] and/or toxin degradation [59]. In our collection, the percentage of cytotoxic strains was higher than those reported by Choma et al. [23]; Jan et al. [24]; Stenfors et al. [25] and Techer et al. [36]. However, the comparison with the results obtained by other authors is difficult because there is a wide diversity in the methods used to test cytotoxicity (various cellular types, incubation times and temperatures, etc.) and the variety of tested foods (milk, vegetables or laboratory collections).

Based on various cellular assays, cytotoxic activity of CytK, NHE and HBL was proven in vitro in previous studies [18, 60–63]. Enterotoxin T showed no cytotoxicity and could probably not contribute to food poisoning [13].

Several studies reported that none of the virulence factor was able individually or in combination to fully explain the cytotoxic potential of *B. cereus* group bacteria [22, 61, 64, 65]. In contrast, our study confirmed that different combinations of enterotoxin genetic determinants are significantly associated to the cytotoxic potential of the bacteria. Therefore, the combined and possibly synergistic action of multiple toxins can probably explain the diarrheal syndrome related to *B. cereus* group bacteria.

Among the strains that possess all of the HBL genetic determinants, the NHE genes, *bceT* or *cytK*, were found in both toxic as well as non-toxic strains. Therefore, as reported by Gilois et al. [66], the presence of a virulence gene does not guaranty the production and secretion of the corresponding protein. Several studies reported that the importance of the enterotoxins is determined by their expression levels and combinations, which is strain dependent. For example, deletion of the *hbl* operon or the *cytK* gene in *B. thuringiensis* strain 407 Cry- did not affect its cytotoxicity [67], while inactivation of the *hbl* operon in *B. cereus* ATCC 14579 reduced the cytotoxic and hemolytic activity [68]. After elimination of *hbl* genes expression the *B. thuringiensis* strain still produced Nhe and CytK, while *B. cereus* ATCC 14579 could only rely on its low Nhe expression.

Virulence gene expression is not only influenced by the genetic characteristics of the strain, but also by environmental parameters, such as the food composition, the pH and the temperature. Food products with a neutral to alkaline pH, high water and starch content and an intermediate glucose concentration pose a potential threat, as their nutrient composition stimulates enterotoxin and cereulide expression in the food and/or in the small intestine [15]. The inhibitory effect on enterotoxin production might be indirect due to growth inhibition of *B. cereus* cells. Both, mesophilic and psychrotolerant *B. cereus* can produce diarrhoeal and emetic toxins. Sometimes, more emetic toxin is strains produced at lower temperatures (12–15 °C) than at 30 °C if more incubation time is granted [69]. Higher incubation temperatures (30–32 °C) generally yield higher enterotoxin concentrations, both for psychrotrophic as mesophilic strains [59]. However, some strains show similar toxin production at high (32 °C) and low (10 °C) temperatures for cultures in BHI with similar biomass [70]. Future studies investigating the effect of environmental parameters on the expression of enterotoxin genes should be conducted by preference under conditions mimicking the human gastrointestinal environment, because the enterotoxin production in food before consumption is generally not considered a major concern. Indeed, these enterotoxins are probably completely inactivated by cooking and gastrointestinal passage because of their thermolability and their sensitivity to proteolytic enzymes and acid pH [15]. Moreover, Gilois et al. [66] have demonstrated that certain toxins such as CytK are unstable and do not persist more than 2 h in *B. cereus* group culture supernatants. Consequently, further studies should be undertaken to assess the expression as well as the stability of the toxins after secretion.

The antibiotic susceptibility of bacteria is a public health concern. Our study has shown that *B. cereus* group strains exhibited various degrees of susceptibility against the antimicrobial agents tested. The majority of the studied strains was resistant to ampicillin, which is in good agreement with previous studies showing a high resistance of this group to ß-lactam antimicrobials [71–73]. This last property may be correlated to the ability of the strains to synthesis ß-lactamase, enzymes involved in the degradation of the antibiotic [73]. In *B. cereus* group, the production of β-lactamases can lead to resistance even up to the third generation of cephalosporins [74]. Eighty-eight percent of strains were resistant to novobiocin. While, Aklilu et al. [26] showed that all the *B. cereus* group isolates they tested showed resistance toward Novobiocin.

Ampicillin and Novobiocin are widely used in the animal production in Tunisia. Ampicillin is used in the treatment of septicaemias, respiratory and urinary tract infections. It was very important in the treatment of many diseases in a broad range of animal species. Few economical alternatives are available. Novobiocin is used in the treatment of mastitis in the form of intramammary creams. The indiscriminate use of such antimicrobial agents in animal husbandry has been linked to the development and spread of resistant bacteria into the environment and their further transmission to humans via the food chain could lead to serious consequences on public health [75]. In addition, there are also human health concerns about the presence of antimicrobial residues in animal products [76]. With the emergence of antimicrobial resistance, the pathogenicity and virulence of these organisms have increased and treatment options are diminishing and also more expensive. More than 85% of the studied strains were sensitive to tetracyclin. This value is more or less consistent with those reported in literature. Arslan et al. [77] reported, for a collection of 29 *B. cereus* group strains, a susceptibility of 89.7% of the strains to tetracyclin. Conversely, Ankolekar et al. [27] found a resistance to tetracycline in 98% of the tested strains. Since *B. cereus* group bacteria are widespread foodborne pathogens, it is important to emphasize that antimicrobial susceptibility testing allows to screen effective antibiotics that warrant therapy in cases of foodborne illness.

The evaluation of the antimicrobial susceptibility of *B. cereus* group bacteria to a variety of antibiotics allows a better control of these bacteria when they are involved in infectious diseases and subsequently a better protection of human health. In our study, susceptibility to the ciprofloxacin was shown in all the isolates from food. Similar to this, Banerjee et al. (2001) [78] received 100% sensitivity to cipro-floxacin in samples from patients, and other authors obtained the same result in testing sensitivity to ciprofloxacin in samples from food [74, 79]. Sensitivity to ciprofloxacin is confirmed by Jensen et al. (2001) [80] in *B. cereus* strains isolated from agricultural soil in Denmark. Therefore, these data demonstrated that ciprofloxacin is relatively effective against *B. cereus group* strains from different sources *as* single agent.

## Conclusion

This is the first report which assesses the toxigenic potential of *B. cereus* group strains isolated from different food matrices in Tunisia. This study evaluates the sanitary risk potential of *B. cereus* group strains by detecting and profiling virulence genes, as well as by testing their cytotoxic activity on Caco-2 cells and their antimicrobial susceptibility. The results showed that this *B. cereus* group collection has a significant toxigenic potential and could become problematic. In order to prevent food poisoning due to this microorganism, further studies could be devoted to the evaluation of the cytotoxicity potential of the strains in more complex microbial environments such as food products stored under different conditions.

## Data Availability

All data generated or analyzed during this study are included in this published article.

## Abbreviations

BceT: Enterotoxin T
BHI-YE: Brain Heart Infusion +Yeast Extract
*ces*: cereulide synthetase gene
CFU: Colony-forming unit
CLSI: Clinical and Laboratory Standards Institute
CytK: Cytotoxin K
DNA: deoxyribonucleic acid
HBL: Hemolysin BL
NHE: Non-Hemolytic Enterotoxin
PBS: Phosphate Buffered Saline
PCR: Polymerase Chain Reaction
PHE: Public Health England
